# “The clan carries us”: kinship, honor, and the collective enactment of family resilience among Yi households living with HIV in rural Southwest China

**DOI:** 10.1186/s12889-026-27151-4

**Published:** 2026-03-30

**Authors:** Xi Zhang, Qiuyue Shang, Qiuhong Chen, Wenxi Zhong, Jian Tang, Mei Liu, Lingyu Tang, Dan Tan, Jing Cheng, Xianjun Mao, Li Ma, ZhaoLan Yu, Yanhua Chen

**Affiliations:** 1 Department of Pain, The Affiliated Traditional Chinese Medicine Hospital, Southwest Medical University, Luzhou, Sichuan China; 2 School of Nursing, Southwest Medical University, Luzhou, Sichuan China; 3 Luzhou Vocational and Technical College, Luzhou, Sichuan China; 4 Department of Infectious Diseases, The Affiliated Hospital, Southwest Medical University, Luzhou, Sichuan China; 5 Department of Antiviral Therapy, The First People’s Hospital of YueXi County, Liangshan, Sichuan China; 6 Chengdu Fourth People’s Hospital, Chengdu, Sichuan China; 7 Department of Nursing, The Affiliated Hospital, Southwest Medical University, Luzhou, Sichuan China; 8 Department of Critical Care Medicine, The Affiliated Hospital, Southwest Medical University, Luzhou, Sichuan China; 9 Luzhou Traditional Chinese Medicine Hospital, Luzhou, Sichuan China; 10 Department of Nephrology, The Affiliated Hospital of Southwest Medical University, Luzhou, China

**Keywords:** HIV, Family resilience, Kinship/lineage, Stigma, Yi people, Rural Southwest China

## Abstract

**Background:**

While antiretroviral therapy has transformed HIV into a manageable chronic condition, prevailing family resilience frameworks, often grounded in Western individualism, may fail to fully capture how families in lineage-based societies negotiate illness and stigma. In the Yi communities of rural Southwest China, kinship and collective honor (, to *jia zhi*) structure the moral world of everyday life, yet their role in shaping family resilience remains underexplored. This study examined how Yi families in Liangshan experience and enact family resilience, with particular attention to the interplay between kinship obligations, moral meanings of care, and HIV-related stigma.

**Methods:**

A descriptive phenomenological design was employed. Semi-structured, in-depth interviews were conducted with 35 adults from HIV-affected Yi households between January and June 2024. Data were analyzed using Colaizzi’s method to elucidate the essential structure of lived family resilience, utilizing Hill’s ABC-X model and Walsh’s family resilience framework as sensitizing frameworks.

**Results:**

Family resilience emerged not as a fixed individual capacity, but as a relational and morally infused practice of “collective endurance”. Two interrelated experiential domains were identified: (1) Resilience woven within the family, sustained through shared presence, reciprocal care, and intergenerational responsibility; and (2) Resilience negotiated beyond the household, characterized by cautious engagement with healthcare resources while managing community surveillance. Central to both domains was *jia zhi* (lineage-based family identity), which functioned as a double-edged sword: simultaneously sustaining essential belonging while intensifying pressures to conceal HIV to protect collective honor.

**Conclusions:**

Family resilience in Yi households is a collective moral practice anchored in the *jia zhi *system. HIV is perceived as a “genealogical rupture.” While “strategic silence” safeguards lineage honor, it imposes significant costs on individual members, especially women. Nursing and public health interventions should adopt lineage-sensitive models that engage clan elders and reframe treatment as “family protection” to ensure cultural alignment.

**Supplementary Information:**

The online version contains supplementary material available at 10.1186/s12889-026-27151-4.

## Background

Despite the global scale-up of antiretroviral therapy (ART), people living with HIV (PLHIV) in resource-constrained and socially stigmatized settings continue to encounter challenges that affect timely testing, linkage to care, and overall well-being [1, 2]. While global initiatives emphasize structural interventions to reduce stigma, evidence confirms that stigma remains a persistent driver of poor health outcomes [3], and family-level dynamics remain a critical determinant of well-being in many communities [4].

In China, national surveillance data show that by 2024, approximately 1.355 million people were living with HIV (PLHIV), with a national HIV prevalence of 0.06%, and both ART coverage and viral suppression rates have surpassed 95% [5]. Notably, China’s HIV response has been supported by two landmark policies relevant to ART provision: the National Free Antiretroviral Treatment Program, initiated in 2002, which has contributed to a notable reduction in HIV-related mortality among PLHIV; and the treat-all policy adopted in June 2016, which permits all PLHIV to access ART irrespective of their CD4+ T cell count [6].

At the same time, evidence from some rural ethnic minority settings suggests ongoing opportunities to strengthen earlier HIV testing and timely engagement in care. In Liangshan Yi Autonomous Prefecture, home to a large Yi population and an area that has experienced a relatively higher HIV burden compared with the national average, the proportion of individuals diagnosed at an advanced stage remains higher than national estimates, indicating delays in testing and care in some communities [7].

Prior studies also suggest that, following diagnosis, some PLHIV in rural areas experience family tension and may choose non-disclosure due to concerns about stigma extending beyond the individual to the wider kinship group [8]. These experiences need to be understood within local cultural and relational contexts. Among many Yi families, identity and moral responsibility are closely connected to *jia zhi*, a lineage-based kinship system that shapes marriage, inheritance, mutual aid, and collective reputation [9]. Within this moral world, HIV may be interpreted not only as a personal health condition but also as an event with implications for family continuity and collective honor. As a result, HIV-related stigma can be experienced relationally, and illness management may become a shared moral project rather than solely an individual concern [10].

Family resilience, therefore, may be enacted through intergenerational solidarity, kinship-based support, and culturally grounded practices aimed at sustaining cohesion and dignity while navigating stigma [8]. Mainstream family stress and resilience frameworks, including Hill’s ABC-X model and Walsh’s family resilience framework [11, 12], have provided foundational tools for understanding adaptation to crisis. Existing literature on HIV-affected families often prioritizes individual psychological attributes [13] or draws heavily from Sub-Saharan African contexts. However, the social repercussions of the epidemic in Africa have fundamentally differed from those in rural China. In Sub-Saharan Africa, high parental mortality has necessitated a transition toward fragmented family units, such as child-headed or skip-generation households, representing a reactive and functional reorganization of caregiving [14]. 

In contrast, family resilience among ethnic Yi communities in Southwest China is rooted in the *jia zhi* (lineage) system, which serves as a durable moral and organizational framework [15, 16]. This system entails a collective responsibility that manifests through institutionalized support mechanisms, including the mandatory absorption of orphans by extended kin and shared economic risk-sharing. Within this lineage-based infrastructure, resilience is not merely a survival-driven adaptation but an expression of intergenerational continuity and ascribed social obligation [16, 17]. This suggests that resilience frameworks must account for these pre-existing kinship infrastructures rather than assuming a universal model of reactive structural collapse.

In the Yi context, where *jia zhi* shapes not only material support but also moral obligation and collective identity, examining resilience requires attention to the relational ethics and social meanings embedded in kinship life. While the *jia zhi* system has been established as a cornerstone of Yi social organization, existing scholarship has primarily examined its macroscopic functions in community governance and health interventions [15]. However, there remains a critical need for empirical evidence on how these lineage-based norms translate into the daily, lived enactment of family resilience for those navigating the specific challenges of HIV. By providing a micro-level analysis of how kinship obligations influence household adaptation, this study contributes to addressing this empirical gap, offering a more nuanced understanding of resilience that complements broad cultural generalizations.

To address this gap, we conducted a descriptive phenomenological study to explore how Yi PLHIV and family members in Liangshan understand and enact family resilience within their cultural world. Although interviews were conducted with individuals, participants consistently narrated experiences through the perspectives and actions of spouses, parents, elders, and kin, highlighting resilience as a relational and collective process. This study addresses three research questions: (1) How do kinship obligations, HIV-related stigma, and nationally supported HIV services interact to shape everyday family life?; (2) What culturally embedded resources, such as *jia zhi*, collective honor, and traditions of mutual aid, enable or constrain adaptive responses to HIV?; and (3) How might prevailing family resilience models be extended to better account for moral worlds organized around lineage-based kinship and relational responsibility?

By centering Yi lived experience, this study aims to inform culturally responsive HIV interventions that strengthen family-centered support and stigma management while safeguarding confidentiality and dignity, thereby contributing to equitable and context-sensitive health responses for ethnic minority populations in China.

## Methods and materials

### Study design

This study adopted a descriptive phenomenological approach guided by Colaizzi’s (1978) method [18] to describe how Yi families in Liangshan experience HIV-related adversity and enact family resilience in everyday life. Although data were generated through individual interviews, our analytic focus was family life as a relational unit because participants consistently narrated experiences in terms of shared obligations, intergenerational responsibilities, and kinship-mediated meanings associated with *jia zhi* (clan/lineage-based family identity).

Consistent with descriptive phenomenology, we attended closely to participants’ accounts and practiced epoché (bracketing) to minimize the influence of prior assumptions about family stress and resilience. This was operationalized by prioritizing participants’ own moral language over pre-defined psychological categories. For instance, rather than defaulting to a nuclear-centric definition of ‘family,’ we coded the involvement of extended kin as an inherent *jia zhi* obligation rather than an external source of ‘social support.’ This reflexive focus ensured that our findings remained grounded in the Yi cultural logic of relational responsibility, avoiding the bias of Western-centric resilience models.

Mainstream frameworks, such as Hill’s ABC-X model and Walsh’s family resilience framework [10, 11], were not used to guide coding or theme development; rather, they served as sensitizing references during interpretation to contextualize how stressors, resources, and appraisals may operate within extended kinship networks beyond nuclear-family-oriented assumptions.

### Research context and participants

Our fieldwork was conducted from January to June 2024 in Liangshan Yi Autonomous Prefecture, a rural region in Southwest China that serves as a designated focal point for national HIV intervention [7]. The social landscape here is defined by a unique intersection of epidemiological pressure and the traditional *Jia zhi* (lineage-based) system. The feasibility of this study relied on our team’s deep local integration: three co-authors (JT, ML, and LT) are active clinicians within the Antiviral Therapy (ART) department of a county-level hospital. This “insider” positioning allowed us to bridge the gap between clinical services and community inquiry, providing the necessary trust to navigate the sensitivities of HIV while ensuring our engagement remained rooted in Yi cultural norms.

Sampling followed a two-stage process. First, we purposively identified the specific ART clinics where our clinical co-authors practice, as these facilities serve as primary care hubs for a large number of rural Yi families. Within these clinical settings, convenience sampling was then used to recruit eligible participants during their routine follow-up visits. To address potential power imbalances inherent in the provider-patient relationship, we ensured that the clinical co-authors (JT, ML, and LT) focused on logistical coordination and cultural mediation, while the primary interviews were conducted by research team members not involved in the participants’ direct medical care. This separation of roles aimed to minimize social desirability bias and encourage open dialogue regarding sensitive cultural and family experiences.

Eligible participants were adults (≥ 18 years) who (1) had an HIV diagnosis consistent with current national clinical guidelines; (2) were able to communicate in Mandarin or Nuosu (with interpreter support when needed); and (3) provided informed consent. Participants were either people living with HIV (PLHIV) or their primary family caregivers. We interviewed one adult per household; each interview therefore represented a distinct household unit. All eligible individuals approached agreed to participate.

To describe household structure, we categorized households by co-residence patterns: nuclear (parents and unmarried children); stem (parents co-residing with one married child); joint (two or more married siblings co-residing); and single-parent households. This typology is consistent with ethnographic descriptions of family organization in Liangshan.

Data collection continued until no substantively new meanings relevant to the study aim were identified in ongoing analysis discussions, and the research team agreed that the dataset provided adequate depth for phenomenological description.

### Interview guide development

A semi-structured interview guide was developed based on: (1) a review of literature on HIV, family resilience, and kinship; (2) consultation with local healthcare staff regarding feasibility and cultural appropriateness; and (3) the phenomenological aim to capture participants’ lived experiences of family life with HIV. The guide was pilot tested with two individuals, and minor refinements were made to improve clarity and cultural resonance (e.g., replacing ‘coping’ with ‘how your family carries the burden’). Interview prompts explored: (1) family responses to HIV diagnosis; (2) perceived impacts on family relationships and daily life; (3) support and pressures from kinship networks (including *jia zhi*); (4) experiences of stigma and disclosure decisions; (5) engagement with healthcare services and publicly funded support; and (6) changes over time and hopes for the future. Open-ended questions and follow-up probes were used to allow narratives to unfold in participants’ own terms.

### Data collection procedures

Interviews were conducted by the first author, a female master’s student in public health nursing at the time of data collection, who had received extensive training in qualitative interviewing. She was familiar with the local sociocultural context but had no prior relationship with the participants before the study. When Nuosu interpretation was required, a trained local interpreter assisted with translation. To reduce social desirability and power-related influences, the interpreter’s role was limited to language support; the interviewer led all questions and probes.

Interviews were conducted in private rooms at county-level ART centers to ensure confidentiality and comfort. Prior to each interview, participants were informed about the study purpose, voluntary participation, and confidentiality protections, and consent was obtained. Interviews lasted 20–50 min, were audio-recorded with permission, and supplemented by field notes capturing context, pauses, and emotional cues. Interviews were paused or discontinued if distress was observed. Transportation costs were reimbursed; no additional incentives were provided.

### Data analysis

Audio recordings were transcribed verbatim within 24 h by two bilingual researchers and checked against recordings for accuracy. Transcripts were anonymized using numerical identifiers (P1-P35), and potentially identifying details were removed. The analysis was supported by a multidisciplinary team with expertise in public health, nursing, and clinical HIV care. The project was led by experienced scholars and clinical experts (including YC, ZY, and XZ), and all team members responsible for data collection and analysis (XZ, QS, QC, and WZ) underwent systematic training in phenomenological inquiry before the study began. This structure allowed for a vital “insider-outsider” dynamic: while the co-authors based in Liangshan (JT, ML, and LT) utilized their long-term clinical presence in local ART clinics to bridge cultural gaps and build trust, the core analysis team maintained the necessary critical distance. During the process, we held weekly meetings to discuss our reflexive memos and consciously “bracket” our clinical assumptions regarding individual treatment adherence. This reflexive practice ensured that the interpretation of resilience remained anchored in the participants’ internal logic of “collective survival” and “lineage honor” rather than being filtered through a purely biomedical lens.

Data analysis followed Colaizzi’s structured descriptive phenomenological method to enhance analytic transparency [18]. Steps included: (1) repeated reading for immersion; (2) extraction of significant statements; (3) formulation of meanings; (4) clustering meanings into themes; (5) integration into an exhaustive description; and (6) articulation of the essential structure of family resilience. To enhance credibility, we conducted member checking with eight participants by sharing a summary of key themes and inviting feedback on resonance and accuracy; feedback was used to refine wording and clarify meanings where needed. Throughout analysis, the research team maintained reflexive memos documenting positionality, bracketing practices, and analytic decisions to strengthen dependability and confirmability.

### Ethical approval and consent to participate

This study was approved by the Biomedical Ethics Committee of Southwest Medical University (Approval No.: SWMUIRBTX-202403-0006) and conducted in accordance with the Declaration of Helsinki. Given potential literacy limitations and the sensitivity of HIV-related documentation in small communities, the ethics committee approved verbal informed consent. Consent procedures were conducted in Mandarin or Nuosu, emphasized voluntariness and the right to withdraw without affecting access to care, and verbal consent was audio-recorded and documented. To minimize deductive disclosure risk, all potentially identifying information (e.g., village, clan affiliation, occupation, and highly specific kinship details) was removed during transcription. Data were stored on password-protected, encrypted devices accessible only to the research team. Findings are reported in aggregated and de-identified form. Prior to data collection, the research team consulted with local health administrators regarding culturally appropriate procedures and participant protection; these consultations focused on study processes and did not involve any participant identities or recruitment lists.

## Results

Findings are presented as interwoven dimensions of lived experience rather than discrete causal factors. Two overarching experiential domains were identified: (1) Resilience Woven Within the Family, describing how households sustained cohesion through shared resources, relational bonds, and morally grounded ways of being; and (2) Resilience Negotiated Beyond the Household, capturing how households navigated healthcare engagement, publicly funded support, and community stigma within lineage-based expectations. These domains were mutually constitutive, reflecting resilience as a relational achievement embedded in both intimate family life and broader socio-moral networks. Table 1 summarizes the core constituents of each domain and illustrative phenomenological expressions.

**Table 1:** Experiential domains and constituents of family resilience among Yi households living with HIV

Domain	Constituent	Description
Resilience Woven Within the Family	Shared Household Resources	Practical economic adjustments, emotional presence, and shared knowledge used to sustain everyday life
	Relational Bonds	Spousal solidarity, parent–child ties, and intergenerational reciprocity
	Morally Grounded Ways of Being	Ways of understanding, enduring, and acting framed as responsibility to family and kin
Resilience Negotiated Beyond the Household	External Support	Community ties and publicly funded services as sources of assistance
	Sociocultural Context and Stigma	Tensions between lineage-based expectations, anticipated/experienced stigma, and changing community norms
	Healthcare Encounters	Treatment access and relationships with healthcare workers as sources of reassurance and hope

### Participants

The sample comprised 35 participants (mean age = 50 ± 14 years; 22 male, 13 female), including 28 people living with HIV and seven family members (e.g., parents, spouses). Each participant represented a distinct household. Most participants reported monthly household incomes below ¥2,000 (57.1%). Household structures included nuclear (*n* = 11, 31.4%), stem (*n* = 16, 45.7%), joint (*n* = 4, 11.4%), and single-parent (*n* = 4, 11.4%) households. Reported routes of infection included heterosexual contact (68.6%), mother-to-child transmission (14.3%), and injection drug use (17.1%). In 62.9% of households, one family member was living with HIV, while 20.0% reported two members and 17.1% reported three or more. Participant characteristics are summarized in Table 2.

**Table 2:** General information of the study population (*n* = 35)

Characteristics		*N* (%)
Gender	Male	22(62.86%)
	Female	13(37.14%)
Age (years)	Young(18–49)	18(51.43%)
	Older(≥ 50)	17(48.57%)
Type of participants	Infected person	28(80.00%)
	Family members	7(20.00%)
Type of family	Nuclear Family	11(31.43%)
	Stem Family	16(45.71%)
	Joint Family	4(11.43%)
	Single-Parent Family	4(11.43%)
Monthly household income	below ¥2,000	20(57.14%)
	¥2,000 to ¥4,000	4(11.43%)
	above ¥4,000	11(31.43%)
Mode of infection	Drug injection	6(17.14%)
	Heterosexual transmission	24(68.57%)
	Mother-to-child	5(14.29%)
Number of existing infections in the family	1	22(62.86%)
	2	7(20.00%)
	3–4	6(17.14%)

### Resilience woven within the family

Participants commonly described resilience not as an individual attribute but as a collective practice embedded in everyday household life. This domain comprised three interrelated constituents: shared household resources, relational bonds, and morally grounded ways of being.

Shared household resources were often described as practical and improvised. Economic constraints were frequently mentioned, and households adjusted routines to sustain care and daily living:

*“After falling ill*,* I couldn’t work outside… I took temporary jobs nearby*,* but my income dropped by more than half.”* (P3, male, 52)

Emotional support was often framed as enacted through steady presence rather than explicit verbal reassurance:

*“We love her just as before and often comfort our sister. Now her mood and health are better.”* (P19, female, 35)

Health-related knowledge was sought to reduce fear and protect family members, rather than to achieve technical mastery:

*“We learn about HIV from short videos online and doctors. Knowing we can protect our family reduces our fear.”* (P23, male, 40)

Relational bonds provided a moral scaffold for endurance. Spousal relationships were described as sources of mutual anchoring:

*“My wife and I were both diagnosed. We comfort each other*,* face difficulties bravely*,* and focus on getting through today.”* (P8, male, 47)

At the same time, participants also described relationship ruptures shaped by stigma and distancing:

*“My son and daughter-in-law cut contact… I’m not allowed to speak to him.”* (P26, female, 63)

Intergenerational co-residence sometimes mitigated these disruptions, with elders described as providing caregiving and guidance:

*“My mother-in-law helps care for the children… and sometimes gives money.”* (P21, female, 38)

Morally grounded ways of being shaped how adversity was understood and confronted. Cognitive shifts from fear toward acceptance were frequently described in family terms:

*“At first*,* we were terrified… Now*,* the whole household has adapted to living with it.”* (P10, male, 44)

Participants framed emotional steadiness as linked to responsibilities toward children or elders:

*“But then I thought: my children need me. I must stay strong and take every pill.”* (P7, female, 50)

Behavioral change was described as relational care rather than solely self-discipline:

*“I quit smoking and drinking— to lighten the burden on my household.”* (P6, male, 41)

### Resilience negotiated beyond the household

While the household was a primary site of care, participants also navigated a broader sociocultural context in which support, constraint, and healing co-existed.

External support was described as coming from both community relationships and publicly funded services. Some participants described neighborly ties as sustaining a sense of belonging:

*“We still eat and drink with neighbors. They comfort us and offer help.”* (P2, male, 58)

Participants also described how publicly funded HIV services reduced financial burden and supported treatment access:

*“Medication is free now—my body and mind have improved.”* (P4, female, 33)

*“We receive free annual check-ups.”* (P12, male, 49)

Sociocultural context and stigma shaped resilience ambivalently. Jia zhi was described as fostering collective responsibility while also intensifying concerns about reputation and disclosure:

*“To avoid shame for our clan, we stay home and skip gatherings.”* (P13, female, 60)

Stigma concerns extended into schooling and social participation:

*“My granddaughter stopped school—she fears classmates will find out she takes HIV medicine.”* (P19, female, 35)

Some participants also described gradual shifts in community attitudes, often in connection with sustained health education by local clinicians:

*“The village doctor kept explaining… They never discriminated…”* (P31, male, 55)

Healthcare encounters were commonly described as transforming despair into possibility. ART was associated with restored health and a renewed sense of agency:

*“This medicine works well… there’s hope for life.”* (P17, female, 42)

Healthcare workers were sometimes described as trusted intermediaries who supported families in navigating both biomedical care and everyday concerns:

*“Our village doctor came home… Without that, we wouldn’t have made it.”* (P29, male, 51)

### The essential structure of Yi family resilience

Across all accounts, the essential structure of family resilience can be summarized as follows:

*“Family resilience was enacted as collective endurance grounded in jia zhi—a lineage-based moral framework through which belonging, obligation, and honor were understood—sustained through everyday caregiving within the household, negotiated through cautious engagement with healthcare services and publicly funded support, and continually adjusted while managing stigma and maintaining family dignity”*

This essential structure reflects not a set of measurable traits but a lived moral world in which resilience was described as duty, dignity, and quiet perseverance—being together in the face of uncertainty.

## Discussion

In Liangshan Yi communities, an HIV diagnosis transcends individual health management to fundamentally disrupt the relational and moral foundations through which families organize everyday care. From a nursing and family health perspective, our findings suggest that family resilience in this setting is less a linear trajectory of recovery or a set of individual coping capacities than an ongoing, morally grounded practice of “collective endurance.”

While Hill’s ABC-X model [11] provides a foundational understanding of family adaptation to crisis, it is primarily designed to capture the homeostatic processes of nuclear family units. In the Yi context, however, our findings suggest that the “Crisis” (X) is experienced not merely as a functional stressor, but as a potential rupture in genealogical continuity. Furthermore, our data invite a critical reconsideration of Walsh’s framework [12], which identifies “communication clarity” as a core pillar of resilience. Among Yi families, resilience is often enacted through intentional opacity and emotional containment rather than individual self-efficacy [19].

We interpret “strategic silence” not as a lack of coping resources or passive suppression, but as a sophisticated relational strategy that prioritizes the moral survival of the *jia zhi* (lineage) over individual psychological transparency. As one participant poignantly noted, “We cannot cry openly; our tears must fall inside the house.” This active, protective strategy is designed to contain the crisis within the safety of the household, thereby safeguarding the family’s collective dignity and reputation. Consequently, this study broadens existing theory by moving from an individualistic-expressive focus to a more collective-existential understanding of resilience.

### The paradox of lineage: support and constraint

*Jia zhi* emerged as the central mechanism of this resilience, functioning as a complex “double-edged sword.” On one hand, the moral obligation inherent in *jia zhi* ensured survival through reciprocal practices. Participants described how adult children working away from home contributed financially, and how elders took on caregiving roles for grandchildren, reinforcing belonging when HIV threatened social exclusion [20–22]. On the other hand, this same kinship logic imposed strict behavioral constraints. To maintain *mianzi* (face) and collective honor, families often engaged in strategic silence or selective withdrawal to mitigate the risk of family-wide stigma. Our findings regarding the persistent fear of disclosure align with systematic reviews indicating that perceived stigma remains a barrier to quality of life even in the era of free ART [23].

Notably, while HIV is widely recognized as a “biographical disruption” to personal identity [24], our findings suggest that Yi participants experienced it more as a “genealogical rupture.” Unlike the individual-centered disruptions documented in Western literature, HIV here threatens the intergenerational transmission of the family name, property, and ancestral obligations. As one participant poignantly shared: “*If I cannot have children*,* my father’s line ends with me.*” This anxiety transcends personal mortality, reflecting a collective dread that the virus will extinguish the *jia zhi*’s biological and moral continuity.

However, this resilience is purchased at a high internal cost. The collective expectation of secrecy often places disproportionate burdens on female members, whose invisible labor and silent endurance sustain the lineage at the expense of their own well-being [25]. Furthermore, this lineage-based support has clear boundary conditions. In contexts of severe stigma or intra-family conflict, the *jia zhi*’s protective function can fracture fundamentally, shifting from a source of support to a mechanism of exclusion to safeguard the clan’s broader reputation [26]. Recent evidence confirms that such tensions create substantial caregiver burden [27] and that stigma-related attitudes can significantly impede health service utilization in rural Sichuan [28]. These outcomes reveal the stark structural limits of informal kinship networks and underscore the need for structured, family-based institutional interventions to stabilize and supplement traditional support systems [29].

### The role of healthcare and cultural meaning

Engagement with healthcare services was frequently described as dignity-affirming, particularly through access to free ART, consistent with findings reported in other Chinese contexts [30]. However, the psychosocial benefits of these services were contingent upon relational safety. Where community stigma was high, the fear that visiting a clinic might lead to inadvertent disclosure often triggered heightened vigilance and social distancing, undermining the therapeutic alliance [31].

Notably, some participants described utilizing digital platforms (e.g., short videos) to access health information. This aligns with recent studies highlighting the growing role of short video consumption in bridging health literacy gaps and improving mental health in rural China [32]. Furthermore, local spiritual traditions influenced meaning-making; Bimo-related practices often framed illness as a life event requiring moral repair rather than individual failure, aligning with phenomenological perspectives on illness as a lived relational experience [33, 34].

### Temporal evolution of lineage-based support

Although this study identifies the structural pillars of kinship support, our interpretive analysis suggests that family adaptation operates as a fluid process rather than a static state. During the initial crisis following an HIV diagnosis, the *jia zhi* functions primarily as a protective unit, prioritizing strategic silence to forestall immediate social rupture and preserve collective reputation. As the condition transitions into a manageable chronic illness through sustained antiretroviral therapy, the focus of collective effort tends to pivot toward long-term caregiving arrangements and the incremental calibration of disclosure within trusted kinship circles. These dynamics are further complicated by generational shifts in cultural identity. Younger family members, who increasingly leverage digital health literacy [32], may navigate the tensions between privacy and health-seeking differently than family elders who remain centered on traditional moral repair. These temporal and intergenerational dimensions indicate that lineage-based responses are not fixed cultural traits but are instead responsive practices that recalibrate across clinical and social trajectories.

### Implications for public health and nursing practice

These cultural dynamics have direct implications for public health interventions in Liangshan. First, intervention delivery: Given the centrality of lineage, standard individual counseling may be less effective. We recommend exploring family-centered models that include culturally authoritative trusted elders or “prestige figures” within the clan (e.g., the Bimo or clan heads) as gatekeepers to facilitate health education, provided confidentiality is strictly negotiated. Second, stigma reduction: Since stigma is experienced as a threat to collective honor (*mianzi*), anti-stigma campaigns should shift focus from “individual rights” to “family protection.” Messaging that frames HIV treatment as a way to “preserve the lineage” and “protect future generations” may align better with local values than narratives focusing solely on individual well-being. Finally, nursing care must accommodate “strategic silence.” Rather than pressuring patients for full disclosure as a sign of acceptance, clinicians should respect selective non-disclosure as a functional coping mechanism that safeguards family social capital. This approach reflects principles of cultural humility and relational ethics [35], prioritizing the safety of the patient’s social world over rigid adherence to biomedical disclosure norms.

Ultimately, this study captures a shift from individual-centered resilience toward a form of “collective endurance” deeply embedded in the *jia zhi* system. By recognizing strategic silence as a proactive protective mechanism rather than a clinical barrier, our findings offer a different perspective on how collectivist norms shape HIV adaptation. This reframing illustrates that resilience in minority families often follows a logic of genealogical survival, providing a grounded basis for developing more culturally humble and lineage-sensitive health interventions.

### Study strengths and limitations

This study offers an in-depth descriptive phenomenological account of how Yi households living with HIV in Liangshan experience and enact family resilience. By focusing on participants’ lived narratives, the study conceptualizes resilience not as a fixed trait or outcome but as a relational and morally grounded practice enacted through everyday caregiving, selective concealment, and kinship-based obligation, with *jia zhi* (clan/lineage-based family identity) providing an important interpretive context. These findings contribute to nursing and family health scholarship by illustrating how resilience may be enacted collectively and embedded in culturally situated meanings of care, dignity, and belonging.

Several limitations should be noted. First, we interviewed one adult per household; thus, findings represent participants’ perspectives on family life rather than multi-voiced within-household accounts. Although participants often spoke in collective terms and described shared moral imperatives (e.g., “our tears must fall inside the house”), this design may under-represent divergent viewpoints, unspoken tensions, or gendered and generational differences in caregiving and emotional labor. Future studies could incorporate multi-member household interviews and/or ethnographic approaches to more fully capture intrafamilial dynamics.

Second, participants were recruited through township ART clinics, which may under-represent households less connected to care, including those who have delayed testing, experienced interruptions in treatment, or face stronger stigma-related barriers to service engagement. While interpreter support and confidentiality safeguards were used when needed, social desirability bias related to stigma and privacy concerns cannot be fully ruled out.

Third, this study’s focus on a single rural prefecture with unique lineage-based kinship and spiritual traditions may influence the direct generalizability of the results. To address this, these findings should be understood in relation to global scholarship on collectivism and HIV. Although the *jia zhi* system is a specific cultural institution of the Yi people, its underlying functional mechanisms such as collective survival strategies, stigma-driven concealment, and gendered disparities in caregiving align with observations in other kin-centered societies. While certain dynamics like the absolute moral primacy of lineage continuity are deeply rooted in local Yi traditions, other observed processes offer broader theoretical relevance. Specifically, the dual role of kinship as both a primary resource and a restrictive constraint, along with the use of strategic silence to safeguard family honor, likely reflects fundamental patterns of family adaptation in high-stigma settings worldwide. Ultimately, while transferability remains contingent on contextual similarity, these insights provide a comparative framework for future cross-cultural research to distinguish between unique ethnic resilience and universal family responses to chronic illness.

Finally, interviews were conducted at one time point, which limits our ability to observe how resilience practices evolve over time in relation to changing family circumstances and longer-term treatment trajectories. While we have interpretively discussed the potential temporal shifts across the illness course, the cross-sectional nature of our data cannot fully capture the fluid, process-oriented nature of resilience. Future longitudinal qualitative research would be invaluable for examining how these kinship dynamics fluctuate across the life course and for informing the design of interventions sensitive to different stages of the illness experience.

Taken together, these limitations do not negate the credibility of the findings; rather, they delineate the scope of inference and highlight priorities for future research to further develop and test culturally responsive, family-centered approaches to HIV care.

## Conclusions

This study reveals that among Yi households in rural Liangshan, family resilience in the context of HIV is not merely a collection of individual psychological traits, but a shared, morally anchored practice of “collective endurance” deeply embedded in the *jia zhi* (lineage) system. Our findings suggest that an HIV diagnosis is experienced less as a private biographical disruption and more as a potential genealogical rupture, threatening the biological and moral continuity of the family line.

The research identifies a unique cultural logic where “strategic silence” and “intentional opacity” function as proactive relational strategies to safeguard collective honor (*mianzi*) and preserve social capital against structural stigma. However, this study underscores that such resilience is often maintained at a significant internal cost, frequently placing disproportionate emotional and caregiving burdens on female members. While the *jia zhi* system provides a robust informal safety net, it functions as a “double-edged sword” that can shift from a source of support to a mechanism of exclusion to protect the clan’s reputation.

These insights contribute to the broader scholarship by reframing resilience in collectivist settings as a process of genealogical survival. For nursing and public health practice, our findings emphasize the necessity of moving toward lineage-sensitive and culturally humble care models. Interventions should respect the internal logic of minority kinship systems by engaging culturally authoritative figures, such as clan elders, as partners in care. Furthermore, health messaging should be reframed from “individual rights” to “family protection,” aligning HIV treatment with the cultural priority of safeguarding future generations. Ultimately, these findings provide a valuable comparative framework for supporting families in similar high-stigma, kin-centered minority settings.

## Supplementary Information

Supplementary Material 1.

## Data Availability

The datasets used and/or analysed during the current study are available from the corresponding author on reasonable request.
