# Supplementary material for: “The clan carries us”: kinship, honor, and the collective enactment of family resilience among Yi households living with HIV in rural Southwest China
Source: BMC Public Health. 2026 Mar 30;26:1678. doi: 10.1186/s12889-026-27151-4 (PMC13196013; doi:10.1186/s12889-026-27151-4)
Supplement: Supplementary file 1 — Supplementary Material 1. [file 12889_2026_27151_MOESM1_ESM.docx]

**Interview Guide for “The Clan Carries Us”: Kinship, Honor, and the Collective Enactment of Family Resilience among Yi Households Living with HIV in Rural Southwest China**

**Introduction**

**Purpose of the Interview:**

This study examined how Yi families in Liangshan experience and enact family resilience, with particular attention to the interplay between kinship obligations, moral meanings of care, and HIV-related stigma.

**General Information**

**1.Demographic Information:**

Could you please share your age, gender, and your role in the family (e.g., parent, spouse, child)?

What is your current living situation (e.g., alone, with family, in a community)?

**2.Family Structure:**

Could you describe your family structure (e.g., nuclear family, extended family)?

How many members are in your family, and what are their relationships to you?

**3.HIV/AIDS Diagnosis:**

When and how did you or your family member(s) learn about the HIV/AIDS diagnosis?

How did you and your family initially react to the diagnosis?

**Impact of HIV/AIDS on Family Life**

**1.Emotional and Psychological Impact:**

How has HIV/AIDS affected your emotional and psychological well-being?

Have you experienced any feelings of sadness, anxiety, or fear? How have you managed these feelings?

**2.Social and Relationship Impact:**

How has HIV/AIDS affected your relationships within the family?

Have you experienced any changes in your social interactions or community involvement?

**3.Economic Impact:**

How has HIV/AIDS affected your family’s financial situation?

Have there been any changes in employment or income due to the illness?

**4.Healthcare and Treatment:**

Could you describe your experience with healthcare services and treatment for HIV/AIDS?

How has the treatment impacted your daily life and family responsibilities?

**Family Resilience and Coping Strategies**

**1. Personal Resources:**

What strategies have you employed to manage the stress associated with HIV/AIDS?

Have you sought information or education about HIV/AIDS? How has this assisted you?

**2. Family Resources:**

How has your family provided mutual support during this period?

Have other family members offered emotional or practical assistance?

**3. Social and Community Resources:**

Have you received support from your community, friends, or other social networks?

How has government policy or healthcare support aided your family?

**Future Outlook and Expectations**

**1. Personal and Family Goals:**

What are your personal and familial aspirations for the future?

Are there specific goals or plans you have to enhance your quality of life?

**2. Support Needs:**

What kind of support do you believe would be most beneficial for your family moving forward?

Are there particular resources or interventions you think would be advantageous for families dealing with HIV/AIDS?

**Conclusions**

**Final Thoughts:**

Is there anything else regarding your experience that you would like to share that we haven't covered?

Do you have any recommendations for how we can better assist families affected by HIV/AIDS?

**Notes for Interviewers**

**Probing Questions:** Utilize open-ended questions and encourage detailed responses. Follow up with additional probing questions if further information is required.

**Empathy and Sensitivity:** Conduct the interview with empathy and sensitivity, recognizing the emotional sensitivity of the topic.

**Cultural Sensitivity:** Be mindful of cultural differences and the significance of family and community in the context of HIV/AIDS.

**Confidentiality:** Assure participants of the confidentiality of their responses and the voluntary nature of their involvement.
